# Further examining how animals weigh conflicting information about reward sources over time

**DOI:** 10.1007/s10071-025-01982-x

**Published:** 2025-07-30

**Authors:** Jack Van Allsburg, Timothy A. Shahan

**Affiliations:** Department of Psychology, Utah State University, Logan, UT USA

**Keywords:** Dynamic averaging, Spontaneous recovery, Choice, Temporal weighting rule, Concurrent schedules

## Abstract

Spontaneous recovery of choice is a behavioral phenomenon where a delay period (without new experience) elicits the recovery of a preference consistent with a previous distribution of rewards, rather than the most recently experienced distribution of rewards. On short timescales (< 48 h), the occurrence of spontaneous recovery of choice has been effectively predicted by the Temporal Weighting Rule. However, previous study of this phenomenon over longer timescales (> 48 h) has found results inconsistent with model predictions. The present experiments investigated three potential explanations for these results: (1.) whether time’s passage alone causes animals to revert to random exploratory behavior; (2.) whether time’s effect on behavior is moderated by experience of volatility in rewards during training; and (3.) whether a drift toward random exploratory behavior produced by time’s passage can be distinguished from the effect of spontaneous recovery of choice. Subjects experienced varied reward conditions in a concurrent choice procedure before preference between options was evaluated at various test delays. Obtained results ruled out these first two explanations, but were inconclusive in distinguishing the effects of a drift toward random exploratory behavior from the effect of spontaneous recovery of choice. Limitations and directions for further investigation are discussed.

Modeling the way that animals make decisions over time in changing conditions remains an important and unresolved topic in the study of choice. Holding other relevant factors (like predation risk) equal,^1^ animals are theorized to use a choice strategy evolutionarily selected to maximize food intake per unit time (review of optimal foraging theory in Pyke 2019). A variety of model approaches have been put forth as potential descriptions of how animals may achieve this goal. Many of these models successfully describe the behavior of real animals (or provide effective models of how an agent might optimize its behavior) under various conditions. However, the actual learning and decision-making mechanisms that underpin these strategies are not fully understood, especially for animals making decisions over longer timescales. Building on a previous investigation of these models (Van Allsburg and Shahan 2024), the current study describes an experimental investigation of possible explanations for why these models failed to predict the behavior of rats in a concurrent choice free-operant procedure.

The constant variability of natural environments presents a challenge for foraging animals. When individuals allocate the limited time they have available for foraging, information relevant to their decision-making, like food availability in a given location, is subject to variation over time—adding time-sensitivity to the information that guides their behavior (McNamara and Houston 1987; Dow and Lea 1987) and imposing limitations on the proportion of relevant information that can even be tracked (McNamara and Houston 1980). This variability and incompleteness means that effective choice strategies must be temporally dynamic, reflecting not just *what* is true in the environment, but *when* it is (or was) true (Kacelnik and Brunner 2002; Owen-Smith 2008). The question of how an animal, or any agent, integrates past experience to make optimal decisions over time bears relevance for a wide variety of fields, from artificial intelligence and reinforcement learning to behavior analysis and behavioral ecology. The universality of this question has led to models from a variety of theoretical foundations.

One common approach to modeling choice behavior over time essentially constitutes different ways of making the matching law temporally dynamic. The matching law (Herrnstein 1961) describes a quantitative relationship often found in studies of concurrent choice (where subjects choose between two or more behavioral alternatives). The relationship is simple—relative behavioral allocation to each option equals the relative rate of reward associated with that option (review in Houston et al. 2021). In a two-option scenario (Eq. 1) *R*_*i*_ signifies the reward associated with a given option and *B*_*i*_ signifies behavior allocated to an option.

1 $$\frac{{R_{1} }}{{R_{1} + R_{2} }} = \frac{{B_{1} }}{{B_{1} + B_{2} }}$$

This law was notably revised to account for various deviations from matching by the generalized matching law (Baum 1974; Eq. 2). In this revision, the ratio of behavior allocated to one option, relative to another option, is set equal to the ratio of their reward rates, multiplied by a bias parameter (*k*; accounting for preference between options independent of reward ratio) and raised to the power of a sensitivity parameter (*a*; accounting for the extent to which behavioral allocation is controlled by the reward ratio between options).

2 $$\frac{{B_{1} }}{{B_{2} }} = k\left( {\frac{{R_{1} }}{{R_{2} }}} \right)^{a}$$

The matching law, and extensions of it, have enabled the effective description of steady-state behavior (behavior under invariant reward conditions) in a wide variety of experimental situations and vertebrate species (review of empirical study in Davison and McCarthy 1988). In fact, matching is so ubiquitously found in vertebrates and so readily adopted by experimentally naïve subjects as a choice strategy that some have suggested it may be innate to vertebrate cognition (Gallistel et al. 2007).

Many recent models of choice built on the matching law commonly replace reward (or reinforcement) rate with an omnibus construct of “value,” a dimension representing a common currency by which different behavioral alternatives are compared and quantifying the expected consequences (such as rewards or punishments) associated with selecting a given alternative (following the concatenated matching law; Baum and Rachlin 1969). As such, “value” typically subsumes various characteristics of reward or punishment associated with one option or another, such as the rate, magnitude, immediacy, or quality of rewards or punishments linked to that alternative. Further, the “standard model” of value-based choice in computational neuroscience typically comprises two processes—the tracking of value in the environment over time and the computation of choice policy (i.e., a function describing the probability of different behavioral alternatives being chosen) from those tracked valuations (review in Glimcher 2014). In other words, the matching law acts as a “decision rule” which takes the valuations of different options as inputs to determine how behavior will be allocated in any given moment. This creates some complexity in interpreting the influence of time on behavior for these models: time could influence the valuation process, the decision process, or both processes simultaneously.

Models employing the matching law as a decision rule often assume that the influence of time is reflected in the valuation process, such that past experience is discounted over time. This notion of progressively discounting past experience follows from empirical findings that animals tend to rely on more recent experience in making decisions, when such experience is available (Cowie 1977; reviewed in Stephens and Dunlap 2017). This process could be linked to memorial decay (more distant memories are more difficult to recall and thus bear less weight in decisions), or it may be an adaptive strategy that allows animals to respond to environmental variability (where memories can still be recalled but their psychological weight is based on their age).

The prevailing approach to discounting past experience is an exponentially weighted moving average (EWMA), which is used widely enough to be called the “common model” of dynamic averaging (Lea and Dow 1984). EWMAs are a common statistical tool used in many fields to smooth minor fluctuations and describe longer term trends, and provide effective description of how animals track reward rates in many circumstances (e.g. Killeen 1984). While not always explicitly termed as such, this approach is functionally employed by influential models such as the linear operator model (Bush and Mosteller 1951), and its descendant, the Rescorla–Wagner model (1972), with widespread adoption across reinforcement learning (reviewed in Katahira 2015; Zhang et al. 2013), computational neuroscience (reviewed in Niv 2009), and choice research, generally (e.g. Navarro et al. 2016; Iigaya et al. 2019).

However, because each recursive update of a EWMA repeatedly multiplies past valuations by the complement of the learning rate (see Appendix), all past experience is discounted at an equal exponential rate. Consequently, the passage of time alone cannot change the relative valuation of available options. This poses a problem for describing phenomena such as spontaneous recovery of choice (SRC)—where a delay period (without new experience) elicits the recovery of a preference consistent with prior reward conditions, rather than the most recently experienced distribution of rewards. SRC has been found in various species (Mazur 1995; 1996; Devenport and Devenport 1993; 1994; Devenport et al. 1997, 1998, 2005; Lattal et al. 2003), and may be related to treatment-relevant phenomena like resurgence, a possible mechanism of relapse (Shahan 2020).

The Temporal Weighting Rule (TWR), an alternative approach to valuation, provides a mechanistic account of SRC. The TWR computes valuations differently from a EWMA (see Appendix), weighting past experiences based on their relative recency. As a result, this model discounts past experience hyperbolically, meaning without new experience, the passage of time causes the value of each option to regress to its unweighted average. This model can therefore predict changes in preference with the passage of time, as with SRC, and has done so effectively in experiments less than 48 h in duration (review in Devenport and Devenport 2009).

However, a recent application of these models to choice over longer timescales (such as days or weeks) revealed significant deviations from the predictions of both TWR and EWMA-based models in a free-operant concurrent choice situation (Van Allsburg and Shahan 2024). The preparation used in this study generated data consistent with SRC that were well-described by the TWR in a short (< 2 h) pilot replication of the procedure originally used to validate the TWR (Devenport and Devenport 1994). While this pilot experiment and other previous experiments on the TWR found an orderly regression toward the unweighted average values, the experiments of the previous study (Van Allsburg and Shahan 2024) are more than an order of magnitude longer in duration. In Experiment 3 of that study, animals experienced a long period (14 days) of conditions heavily favoring one option, followed by a short period (2 days) of reversed conditions (heavily favoring the other option), before experiencing one of four test delays, during which they received no new experience with the options. Instead of following the predictions of a simple EWMA-based model (consistent preference for the most recently rewarded option) or the predictions of the TWR (regression toward overall average valuations—increasingly favoring the originally richer option at longer test delays), the data suggested that animals may be simply drifting toward indifferent responding (equal preference for the two options) over time.

This potential drift toward indifference accompanying the passage of time (in test delay) may be linked to the valuation processes employed—but it could also be explained by changes to parameters of the decision rule. For the generalized matching law (Baum 1974), this drift could constitute a decrease in the discriminability of the two options or a decrease over time in sensitivity to their relative values. The former of these explanations is simpler, implying that, over time, animals may just forget which valuation is associated with which option. The latter explanation, by contrast, could be an adaptive strategy that drives exploratory behavior when animals do not have recent experience to guide behavior. In that case, the sensitivity parameter, which dictates the extent to which valuations influence behavioral policy, might decrease to the point that policy does not reflect valuations and options have equal probability of being selected.

Another approach to temporally dynamic choice modeling, explore/exploit models, provide an alternative way to understand this adaptive drift toward exploratory behavior. In these models, the tradeoff between exploring/sampling options or exploiting those options’ rewards is seen as a spectrum between these two modes of foraging, where animals typically employ some balance of the two modes, depending on the situation (review in Mehlhorn et al. 2015; Berger-Tal et al. 2014; Navarro et al. 2016). Common in both reinforcement learning and behavioral ecology, explore/exploit models use the same structure of tracked valuations and behavioral policy computed through a decision rule, but replace the matching law with a softmax function (Eq. 3; Addicott et al. 2017).

3 $$P_{1} = \frac{{e^{{(V_{1} /t)}} }}{{e^{{(V_{1} /t)}} + e^{{(V_{2} /t)}} }}$$

Here, the probability of selecting an option (*P*_*x*_) is set equal to the exponential of the options’ value, multiplied by an inverse temperature parameter (1/*t*), and divided by the exponentials of the values (also multiplied by 1/*t*), for all other options under consideration. Analogous to the generalized matching law’s sensitivity parameter, the inverse temperature parameter of this function dictates the extent to which policy is based on the valuations of different options—with exploitation denoting a policy entirely based on valuations and exploration denoting a policy that ignores valuations and favors options under consideration equally. Recent theoretical work (Gershman 2019) has distinguished between “random” and “directed” exploration as two algorithmic approaches to adjusting this inverse temperature parameter. “Random” exploration denotes an approach where the *total* uncertainty in the environment dictates the stochasticity of choice (i.e. inverse temperature). “Directed” exploration denotes a more sophisticated approach where exploration is based instead on information gain, assigning a “bonus” to specific actions with higher *relative* uncertainty. Unlike the circumstance of interest in the current study, this latter approach applies to situations where uncertainty is explicitly signaled to the agent. The belief-representation and updating process entailed by “directed” exploration algorithms is also applied primarily to trial-based, shorter-length experiments, which the effects of each action selection and its outcomes are reflected in molecular, recursive updates to the choice model. Because the current study investigates the molar proportions of responding over time between options with no signaled degree of certainty, any potential effect of time’s passage on the inverse temperature parameter would be best conceptualized as an increase in total uncertainty, leading to “random” exploration.

The idea that time’s passage might cause a gradual shift toward random exploratory behavior could indeed potentially explain the results observed in the previous study (Van Allsburg and Shahan 2024), and this sort of shift is a specific consideration of explore/exploit models. However, the actual predictions made by explore/exploit models should not differ from the predictions of a model based on the generalized matching law where time exerts a similar effect on the sensitivity parameter. Further, explore/exploit models may still employ a valuation mechanism like the TWR or an EWMA, so their crucial conceptual difference from matching law-based models lies in their manner of policy calculation, for which the matching law holds more empirical support.^2^ Typically, these models fit inverse temperature as a free parameter (e.g. Daw et al. 2006), rather than linking that parameter to some function of time passing. However, since parameters of the valuation functions feeding into the decision rule can affect performance in similar ways to this inverse temperature parameter, a variety of parameter combinations can fit data (Ballard and McClure 2019). This issue with parameter identifiability challenges the extension of such an approach to novel experimental contexts, and complicates the interpretability of such fits.

Regardless, evaluation of different modeling approaches in the light of inconsistent findings at longer timescales provides the foundation for the current study. The three current experiments are manipulations of the basic procedure used in the previous study (Van Allsburg and Shahan 2024) to further investigate the mismatch between the observed preference data and the predictions of the various models under investigation. First, Experiment 1 directly tests the possibility that the passage of time alone causes a drift to indifference. Second, Experiment 2 tests if this drift to indifference accompanying the passage of time might only occur if the conditions of reward change at some point during training. That is, this experiment examines if effect of time passing may be moderated by the degree of volatility in reward conditions. This idea is borne from models which link the degree of volatility in an environment to the rapidity of changes in learning rate—a relationship found in various species (Behrens et al. 2007; Saito et al. 2014; Gallistel et al. 2001; and Piet et al. 2017). Third, Experiment 3 evaluates if the procedure used in the previous study (Van Allsburg and Shahan 2024) has a methodological limitation. A full reversal of preference between early and late tests would have provided compelling evidence that animals are regressing to an unweighted average valuation as predicted by the TWR. However, because animals reverted to a lesser degree than would be predicted by the TWR/sTWR in each experiment, this shift in preference cannot be readily identified as a change in valuations or a change in decision rule parameters (a drift to indifference), as the direction of both these influences would be identical in appearance. Distinguishing between an effect of time on the valuation process or an effect of time on the decision process may inform revisions to current models that could allow them to account for the observed data.

## General methods

### Subjects

Subjects were experimentally naive male Long-Evans rats (~ 72–92 days old), housed individually in a humidity and temperature-controlled colony room, illuminated on a 12:12 h light/dark cycle. Subjects were maintained at 80% of their free-feeding weight and provided ad-libitum access to water. Sessions occurred 7 days/week at approximately the same time each day.

### Apparatus

Ten identical operant chambers (30 × 24 × 21 cm; Med Associates) were housed in light-and sound-attenuating cubicles. Chambers comprised work panels on the left and right walls with a clear Plexiglas ceiling, front door, and back wall. On the left panel, a centered house light provided chamber illumination. On the right panel, two retractable levers were positioned on either side of a food magazine. Med-PC software controlled experimental events and data collection.

### Procedure

#### Overview

Each of the three current experiments examined the relation between test delay and SRC using a between-subjects comparison of preference, exposing all subjects to the same history of rewards during a training phase (each experiment using different reward conditions), followed by one of four test delays, with subjects randomly assigned to the different test delay conditions. Further details consistent in all three experiments are described below, and details specific to each experiment are provided in the corresponding experiment description.

#### Preliminary magazine and lever training

Experiments began with a single session training subjects to retrieve pellets from a magazine. 30 pellets were delivered via magazine on a 60 s variable-time schedule. Each pellet delivery was accompanied by the illumination of the magazine for 3 s. In four subsequent sessions, rats were trained to perform the lever press response. In these lever-press acquisition sessions, the house light illuminated to signal the session’s beginning. Simultaneously, one of the two levers was extended. Each lever press retracted the lever, extinguished the house light, and resulted in a pellet delivery. Each delivery began a 3 s consumption period, during which the magazine was illuminated and the lever remained retracted. After the consumption period, the lever extended again, and the house light was illuminated. Option A and B lever assignment was counterbalanced across subjects so the right-hand lever was Option A for half the subjects, while for the other half the right-hand lever was assigned as Option B. Further, the presentation of levers during training was also counterbalanced, such that half the subjects were trained on the “Option A” lever on days 1 and 3 of lever training, while the remaining subjects were trained on the “Option B” lever on those same days. Subjects were trained on the opposite lever on days 2 and 4 of lever training. Sessions terminated once each subject had earned 100 pellets.

#### Training phase sessions

During training phases, daily sessions began with the illumination of the house light and extension of both levers. In the first session of the first phase, both levers were initially baited such that the first lever press resulted in a pellet delivery. Following this first press, and for all other sessions during training, levers were baited on concurrent VI schedules. All VI schedules were constructed using ten intervals derived from the Fleshler and Hoffman (1962) distribution (see Appendix for full explanation of VI construction). To prevent animals from employing an alternating strategy, a 3 s changeover delay (as in Baum 1982) was employed (following each lever press, there was a 3 s delay before presses on the opposite lever would result in a pellet delivery). As in lever training, the delivery of a pellet initiated a 3 s consumption period, during which both levers retracted and the session timer paused. Following this period, the levers extended again, and the timer resumed. Sessions terminated after 30 min. Training phase lengths and schedules are detailed in each experiment.

#### Test phase sessions

Following the training phase and one of four test delays, subjects were tested for preference during a single session. Subjects were assigned to one of four groups, each corresponding to a different delay: Group 1 was tested with a 1-day test delay (test session occurred on the day after the training phase), Group 2 with a 3-day test delay, Group 3 with an 8-day test delay, and Group 4 with a 32-day test delay. Specifics of group assignment are detailed in each experiment. Test delay lengths were chosen to remain consistent with previous study of how preference changes during test delays (Van Allsburg and Shahan 2024) to allow for direct comparison of performance at each time point. During test delays, animals remained in their home cage and maintained at their current weight. During test sessions, the house light illuminated to signal the beginning of the session, and both levers extended, but neither response was baited. The first 2 min of responding in each test session was used in data analysis to assess initial preference (responses to Option A divided by total responses).

## Experiment 1

Experiment 1 evaluated the stability of preference at different delays following training under invariant reward conditions. This experiment aimed to determine if the passage of time alone was sufficient to cause a drift toward random exploratory behavior. Animals were exposed to a single training phase in which the same reward conditions were always in effect, then tested in groups following the four test delays specified in the general methods section.

### Method

Thirty-eight rat subjects underwent magazine and lever training before a training phase and test sessions, as detailed above (see General Methods). One of the original forty rats was dropped for failing to acquire the lever press response during lever training, and another was dropped during the training phase due to illness. Conditions of reward were not varied during the 14 day training phase: reward proportions favored option A at a ratio of 9:1. This ratio was achieved by baiting option A on a VI-10 s schedule of reward and option B on a VI-90 s schedule of reward. Subjects were assigned to test delay groups using a rank-match process based on the average preference during the last two days of the training phase, such that the 4 rats with the highest preference were assigned to separate groups, followed by the 4 rats with the next highest preference, and so on (Training Phase terminal logit^3^ preference comparison ANOVA: *F*(3,35) = 0.026, *p* = 0.994). The shorter two test session groups (1 and 2) were each assigned 9 subjects, while the latter two test session groups (3 and 4) were assigned 10. Test sessions for each group were conducted following their corresponding delay.

### Results

**Fig. 1:**
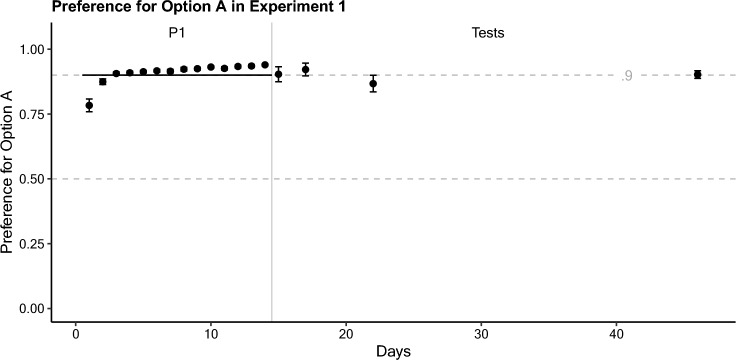
Preference for option A (*P*_*A*_) as a function of time (days) during Experiment 1. Phase 1 is indicated by P1, with a grey line dividing the training and testing phases. Relative reward rate is visualized on the same axis with a black line. Error bars represent the standard error of the mean

Subjects acquired a strong preference for option A (*P*_*A*_) of 0.940 by the end of the training phase of 14 daily sessions (P1; see Fig. 1). This preference was consistent for all four tests (Table 1). While there was minor variance around the programmed reward proportion of 0.9, the overall results suggest that no drift toward indifference occurred following consistent conditions of reward in training. Indeed, an ANOVA comparison of logit preference in test sessions between groups was non-significant (*F*(3,35) = 0.95, *p* = 0.427). Because training conditions were invariant, all models under consideration provided identical predictions at all time-points equal to the reward proportion (*P*_*A*_ = 0.9), consistent with the results. A comparison of their fits would therefore simply reflect their parameterization. Consequently, such a comparison is omitted here.

**Table 1:** Experiment 1 Test Session Data

Test delay	Day	Proportional Preference for A	*SE*	Logit Preference for A	*SE*
1	15	.904	.029	2.55	.306
3	17	.921	.024	2.84	.350
8	22	.867	.032	2.17	.321
32	46	.902	.015	2.34	.185

### Discussion

This experiment aimed to determine if the potential drift toward indifference observed in the previous study (Van Allsburg and Shahan 2024) was simply a result of animals reverting to a random exploratory mode (giving both options equal value) following sufficient passage of time. These results provide clear evidence that such a drift does not occur after consistent conditions of reward during training. At each delay, even 32 days after the end of training, group preference remained near (within a standard error of the mean) of *P*_*A*_ = 0.9, matching the reward proportions during training. Given these results, the observed change in preference with the passage of time in the previous study (Van Allsburg and Shahan 2024) could be linked to the experience of varying reward during training. What remains unclear, from these results, is whether the experience of *any* variance in reward conditions is sufficient to produce a drift to indifference, or if such a drift only occurs under specific orders and lengths of conditions.

## Experiment 2

Experiment 2 evaluated the potential effect of volatility in reward conditions during training on preference at different test delays. In other words, the experiment aimed to determine if variance in reward conditions earlier in training was sufficient to cause a drift toward random exploratory behavior. Animals were exposed to three training phases, in which the first and third phases had the same reward proportions favoring option A, and a shorter, second phase of reversed conditions (favoring option B) occurred in-between. Following this training experience, rats were assigned to one of four test delays and preference was recorded during test sessions.

### Method

Thirty-eight rat subjects underwent magazine and lever training before a training phase and test sessions, as detailed above (see General Methods). Two of the original forty subjects were dropped from the experiment because they did not acquire the lever press response during lever training. The training phase of this experiment was divided into three parts. During the first sub-phase (P1), for 7 days, reward proportions favored option A at a ratio of 9:1. This ratio was again achieved by baiting option A on a VI-10 s schedule of reward and option B on a VI-90 s schedule of reward. Immediately following this first sub-phase was a second sub-phase (P2) with reverse conditions for 2 days, swapping the two VI schedules such that option B was favored at a ratio of 9:1. Finally, there was a third sub-phase (P1’) in which conditions reverted to favoring option A at a ratio of 9:1 for an additional 7 days.

Subjects were again assigned to test delay groups using the same rank-match process as in Experiment 1 based on terminal preference during the last two days of training, except groups were further adjusted to also match average preference during the two days of reversed conditions in the second sub-phase (third sub-phase terminal logit preference comparison ANOVA: F(3,35) = 0.022, p = 0.996; second sub-phase terminal logit preference comparison ANOVA: F(3,35) = 0.001, p = 0.999). The shorter two test session groups (1 and 2) were each assigned 9 subjects, while the latter two test session groups (3 and 4) were assigned 10. Test sessions for each group were conducted following their corresponding delay.

### Results

**Fig. 2:**
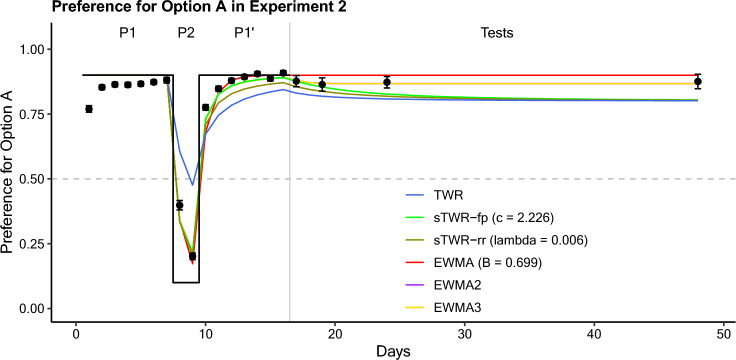
Preference for option A (*P*_*A*_) as a function of time (days) during Experiment 2. Sub-phases 1, 2, and 1’ are indicated by P1, P2, and P1’ respectively, with a grey line delineating the training and testing phases. Relative reward rates are visualized on the same axis with a black stepwise line. Error bars represent the standard error of the mean. Predictions from the models under consideration (model details in appendix) are based on programmed reward rates

Subjects acquired a strong preference for option A (*P*_*A*_) of 0.880 by the end of the first sub-phase of 7 training sessions favoring option A (Fig. 2). Subjects then reversed this preference by the end of the second sub-phase of 2 training sessions under reversed conditions (*P*_*A*_ = 0.201). Finally, subjects recovered a strong preference for option A by the end of the third sub-phase of 7 training sessions under conditions favoring option A (*P*_*A*_ = 0.908).

This terminal preference remained relatively consistent at all four tests (Table 2). All four test groups showed a slight decrease in preference for option A from the end of the training phase, but (after Bonferroni correction) this decrease was only significant for Group 3 (Group 1 paired samples *t-*test: *t*(8) = 1.64, *p* = 0.14, *p*_*corr.*_ = 0.69; Group 2: *t*(8) = 2.67, *p* = 0.028, *p*_*corr.*_ = 0.14; Group 3: *t*(9) = 5.62, *p* = 0.00033, *p*_*corr.*_ = 0.0016; Group 4: *t*(9) = 0.64, *p* = 0.535, *p*_*corr.*_ = 1). Further, the overall results suggest that no drift toward indifference occurred during the test delays. An ANOVA comparison of logit preference during test sessions between groups was non-significant (*F*(3,35) = 0.091, *p* = 0.964, *p*_*corr*_ = 1), suggesting no change over this test delay.

**Table 2:** Experiment 2 Test Session Data

Test delay	Day	Proportional Preference for A	*SE*	Logit Preference for A	*SE*
1	17	.877	.021	2.08	.204
3	19	.864	.026	1.96	.202
8	24	.873	.022	2.06	.205
32	48	.875	.028	2.11	.213

The model comparison (Table 3, model descriptions in appendix) includes the TWR (Devenport and Devenport 1994), the free-parameter sTWR (treating currency, the factor scaling the recencies of past experience, as a free parameter), the reward-rate sTWR (treating currency as a linear function of average reward rate; as in Shahan and Craig 2017), and a model determining relative preference by the matching law as a decision rule with valuations of the two options by EWMA (with learning rate as a free parameter). There are two more versions of this latter model included, with valuations of the two options divided between multiple integrators (EWMAs) using different learning rates (as free parameters) and with additional parameters determining the relative weight given to those integrators over time (also implemented as free parameters). Predictions from the models under consideration were calculated using the programmed rates of reinforcement (see Appendix for further details on the operationalization of time and rewards for different models), and fit to aggregated choice proportions per session, averaged across subjects. The Solver extension in Microsoft Excel was used to fit model parameters by minimizing residual sum of squares (employing the GRG-Non-linear algorithm).

**Table 3:** Experiment 2 Model Fit Comparison (Model Details in Appendix)

Model	Parameter fit	*RSS*	*R*^*2*^	*AIC*	*BIC*
TWR	None	0.207	.664	– 89.39	– 88.40
sTWR-fp	*c* = 2.226	0.039	.936	– 122.50	– 121.50
sTWR-rr	*λ* = .00563	0.053	.913	– 116.35	– 115.36
EWMA	*β* = .699	0.041	.933	– 121.91	– 120.92
EWMA (2 timescale)	*β*_1_ = .834 *β*_2_ = .304 *w*_1_ = .725	0.032	.948	– 122.64	– 119.65
EWMA (3 timescale)	*β*_1_ = .834 *β*_2_ = .304 *β*_*3*_ = 1 × 10^–10^ *w*_1_ = .684 *w*_2_ = .259	0.032	.948	– 118.64	– 113.66

The models are compared in fit using Akaike’s information criterion (AIC) and the Bayesian information criterion (BIC) (review of these measures in Klapes et al. 2018). The overall indication of this fit comparison is inconclusive. While an adequate description of the data may be found in EWMA3, the BIC value for that model is worse, and the apparent support (from this BIC comparison) for sTWR on that metric should be tempered by sTWR’s poor description of the test data, predicting a decelerating decrease that was not observed.

### Discussion

This experiment was intended to determine if variation in reward conditions at any point during training was sufficient to cause a drift toward random exploratory behavior. While there is a perceptible decrease in preference from the end of training to the first test, the relative stability at the same level throughout the testing phase suggests that time did not have an effect on preference during the test delay. One potential interpretation is that the experience of changed conditions may be too temporally distant from the tests to influence the effect of time during the test delay, but the overall finding is unclear. In both Experiments 1 and 2 (and in the previous study, Van Allsburg and Shahan 2024), SRC and a drift to indifference in this experiment would be expected to produce effects on preferences in the same direction. The small change that was observed therefore cannot be clearly linked to either the decision rule or the valuations. Distinguishing these effects therefore serves as the goal of Experiment 3.

## Experiment 3

Experiment 3 attempted to differentiate the effect of SRC (resulting from longer experience with option A being favored) from the effect of a possible drift to indifference, potentially resulting from changed conditions at the end of training. Animals were exposed to two training sub-phases, in which the first sub-phase had reward proportions favoring option A, and was followed by a shorter, second sub-phase of equal reward conditions (favoring both options equally, while holding overall reward rate consistent). Following this training phase, rats were assigned to one of four test delays and preference was recorded during test sessions.

### Method

Thirty-eight rat subjects underwent magazine and lever training before a training phase and test sessions, as detailed above (see General Methods). One of the original 40 subjects were dropped from the experiment because they did not acquire the lever press response during lever training. Another subject was dropped due to lack of responding on the last day of the training phase. The training phase of this experiment was divided into two sub-phases. During the first sub-phase (P1), for 14 days, reward proportions favored option A at a ratio of 9:1, using the same VI schedules as before. Immediately following this first sub-phase was a second sub-phase (P2) with conditions favoring both options equally (1:1) for 2 days, using equal VI-18 s schedules.

Subjects were again assigned to test delay groups using the same rank-match process as in Experiment 2 based on terminal preference during the first sub-phase, and groups were further adjusted based on terminal preference during the last two days of the second sub-phase. (first sub-phase terminal logit preference comparison ANOVA: F(3,35) = 0.014, p = 0.998; second sub-phase comparison ANOVA: F(3,35) = 0.079, p = 0.971). The shorter two test session groups (1 and 2) were each assigned 9 subjects, while the latter two test session groups (3 and 4) were assigned 10. Test sessions for each group were conducted following their corresponding delay.

### Results

**Fig. 3:**
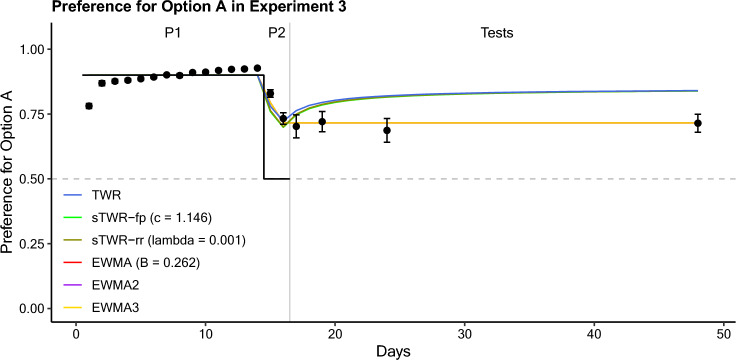
Preference for option A (*P*_*A*_) as a function of time (days) in Experiment 3. Sub-phases 1 and 2 are indicated by P1 and P2, respectively, with a grey line delineating the training and testing phases. Relative reward rates are visualized on the same axis with a black stepwise line. Error bars represent the standard error of the mean. Predictions from the models under consideration (model details in appendix) are based on programmed reward rates

Subjects acquired a strong preference for option A (*P*_*A*_) of 0.927 by the end of the first sub-phase of 14 training sessions favoring option A (Fig. 3). This preference decreased by the end of the second sub-phase of 2 sessions, with conditions favoring both options equally (*P*_*A*_ = 0.732). Again, this terminal preference remained relatively consistent for all four tests (Table 4). While all four tests again showed a slight decrease in preference for option A from preference seen at the end of the training phase, this difference was not significant for any group. (Group 1 paired samples *t-*test: *t*(8) = 1.30, *p* = 0.23, *p*_*corr.*_ = 1; Group 2: *t*(8) = 1.13, *p* = 0.29, *p*_*corr.*_ = 1; Group 3: *t*(9) = 0.56, *p* = 0.59, *p*_*corr.*_ = 1; Group 4: *t*(9) = 0.65, *p* = 0.535, *p*_*corr.*_ = 1). Also, as in Experiment 2, the results suggest that no drift toward indifference occurred during the test delays. An ANOVA comparison of logit preference during test sessions between groups was non-significant (*F*(3,35) = 0.031, *p* = 0.992, *p*_*corr.*_ = 1). The overall results therefore suggest that no drift toward indifference occurred during the test delays—or if such a drift occurred, it may have been counteracted by another influence, such as the spontaneous recovery of choice.

**Table 4:** Experiment 3 Test Session Data

Test delay	Day	Proportional Preference for A	*SE*	Logit Preference for A	*SE*
1	17	.702	.044	0.96	.245
3	19	.721	.039	1.01	.192
8	24	.687	.046	0.92	.265
32	48	.715	.035	0.98	.179

The overall indication of this model comparison (Table 5) favors the account of the single-integrator EWMA model. However, the best fit value of the beta parameter (0.262) for that model is not consistent with the best fit value in the previous study (Van Allsburg and Shahan 2024; Experiment 3): 0.472, or in Experiment 2: 0.699. It should also be noted that the fits of the two- and three-integrator EWMA models are essentially equivalent to the single-integrator EWMA model, as both fits converge on the value of 0.262 for one integrator and assign virtually all the weight to it.

**Table 5:** Experiment 3 Model Fit Comparison (Model Details in Appendix)

Model	Parameter fit	*RSS*	*R*^*2*^	*AIC*	*BIC*
TWR	None	0.064	.543	– 112.86	– 111.86
sTWR-fp	*c* = 1.146	0.063	.553	– 113.28	– 112.28
sTWR-rr	*λ* = 1 × 10^–4^	0.063	.551	– 113.18	– 112.18
EWMA	*β* = .262	0.021	.848	– 134.90	– 133.90
EWMA (2 timescale)	*β*_1_ = .616 *β*_2_ = .262 *w*_1_ = 1 × 10^–10^	0.021	.848	– 130.90	– 127.91
EWMA (3 timescale)	*β*_1_ = .938 *β*_2_ = .674 *β*_*3*_ = *.*262 *w*_1_ = 1 × 10^–10^ *w*_2_ = 1 × 10^–10^	0.021	.848	– 126.90	– 121.92

### Discussion

Experiment 3 was intended to distinguish the effects of SRC and of a drift to indifference over time. The results of this experiment, however, are difficult to interpret, as the stable preference observed during the testing phases may be interpreted as neither effect taking place or both effects having equal, but opposite influence on preference over time.

## General discussion

Taken with the results of the previous study (Van Allsburg and Shahan 2024) the results of these experiments are difficult to interpret within any of the modeling frameworks described thus far. Experiment 1 provides strong evidence that animals behave in a manner consistent with past reward conditions (approximating a matching relationship) at delays as long as 32 days—so long as those conditions are invariant. Any straightforward effect of time that simply drives animals toward random exploratory behavior as test delay increases may be ruled out by this experiment, as animals clearly maintained their relative preference for the duration of the testing phase. The change observed between groups during the test delays in Experiment 3 of the previous study (Van Allsburg and Shahan 2024) might then be interpreted as the effect of time *moderated* by the experience of variation in reward conditions during training. However, in the present Experiment 2, the experience of variation in reward conditions alone (2 days of reversed conditions in the middle of training) failed to produce noticeable changes in preference during the test delay. This second experiment therefore suggests that if a drift to indifference resulting from time passing, the moderating influence of variation in reward conditions is dependent on the specific position/timing of that variation in the training—in other words, this drift might only occur when there is variation at (or near) the *end* of training. Finally, in Experiment 3, we were unable to positively identify or distinguish the effect of a drift to indifference from SRC. Two possibilities are suggested by the data. It is possible that a drift to indifference and SRC both occurred and had approximately equal (but opposite) effects over time, resulting in no discernible change—or that neither of these effects occurred, and preference simply remained static. We will attempt to interpret these results and propose how a model might predict them, as well as describe some methodological limitations of our current experimental preparation.

To explain the conceptual gaps in what we learned from these experiments, we will start by identifying the commonalities between them. First, when choosing between options, subjects acquired relative preferences consistent with the relative rewards associated with each option—i.e., subjects approximated the matching law in their responding. Second, when conditions were changed, subjects adjusted their preferences to match the reward conditions. Third, neither this acquisition nor this adjustment happened immediately, animals had to sample the options before gradually approaching an asymptotic relative preference for each option equivalent to its relative rewards. Fourth, in all three of the current experiments, preference was roughly consistent with the most recent reward conditions at each test delay.

These first two commonalities are straightforwardly described by the matching law. The third commonality can be logically linked to some process of stochastically discriminating the reward rates of options in the environment. This fourth commonality, however, stands in contrast to the experiments of the previous study (Van Allsburg and Shahan 2024). In those experiments, increasing test delays showed a shift from preference reflecting recent conditions to preference, consistent with either SRC or a drift to random exploratory behavior. This begs the question: what differences between the current experiments and the experiments of the previous study (Van Allsburg and Shahan 2024) could explain this inconsistency?

Experiment 1 differed from the previous study (Van Allsburg and Shahan 2024) by omitting variance in training conditions, suggesting variance is necessary to observe changes in preference as test delay increases. Experiment 2 differed from the previous study (Van Allsburg and Shahan 2024) by placing the experience of variance in reward conditions in the middle of training, but found similar test results to Experiment 1, suggesting that the specific timing of this variance within training is relevant—perhaps only recent variance produces changes in preference as test delay increases. Experiment 3 differed from the previous study (Van Allsburg and Shahan 2024) in that the “changed” conditions of reward during training favored both options equally, rather than reversing a strong preference for one option. It should also be noted that animals were slower to adjust their responding when conditions changed in this last experiment, potentially because the change in conditions was less discriminable (Bizo and White 1994; Davison and Jenkins 1985), relative to a full reversion of reward conditions.

A model that could successfully account for these results and the results of the previous study (Van Allsburg and Shahan 2024) would need to link the influence of time to some measure of recent volatility in reward conditions. However, as described before, simple approaches like linking the sensitivity parameter of the generalized matching law to the summed standard deviations of reward rates experienced produce nonsensical predictions, even if that standard deviation is only calculated over a shorter “window” of past experience, or if it is dynamically averaged by a TWR or EWMA.

The difficulty of determining what constitutes “recent volatility” brings up other problems with the operationalization of time for these experiments. Sessions/days are treated as individual timesteps, but the animals are only gaining 30 min of experience within that timestep, with an additional 23.5 h that are not accounted for by the model. If that intersession time is reflected in the decay functions of past experience, the influence of even the previous day declines quickly—leading to infinitesimal valuations for EWMA models and a rapid regression toward overall average valuations for the TWR. The TWR’s support is drawn from experiments that do not include such intersession delays (e.g. Devenport and Devenport 1994; Devenport et al. 1997). It is unclear how this intersession time might affect dynamic averaging models, but this difference could account for the failure of these models at this timescale. Resolving this question might be accomplishable with a (somewhat complex) comparison of how animals behave in such a procedure at various timescales, including conditions where rewards are distributed evenly over sessions of different lengths and conditions where equal length sessions are spaced out by different intersession intervals. Such a comparison, if consistent with the statistical power of the current experiments, would bear a high cost in subjects and time.

This highlights another limitation of this procedure—while a within-subject approach to studying this topic could enable the use of fewer animals, between-subject designs bear a high cost, relative to the amount of meaningful data gained. Each group in these experiments was designed to have 10 subjects. Those 10 subjects, who must be experimentally naïve to control for biases between options, only produce one truly meaningful data point: their group preference at their assigned test delay. The significant tradeoff of effort and cost in animal life required to conduct such large experiments—relative to the amount of useful information such experiments generate—makes it difficult to defend this approach as a sustainable method of studying these questions. While a variety of manipulations to this procedure could continue to get us closer to understanding this problem, the failure of the current experiments to explain the results of the previous study (Van Allsburg and Shahan 2024) suggests that such minor manipulations may be not be the optimal approach. Novel, alternative means of investigation could be effective, such as employing invertebrate species models or some yet-undetermined preparation that would allow for repeated experimentation on the same subjects.

There are also alternative choice model approaches which could be adapted to this context, despite their currently limited relevance. These “policy gradient” models, largely developed in the reinforcement learning literature, eschew valuations entirely, and are argued to provide more parsimonious accounts of decision-making under many conditions (review in Bennett et al. 2021). In these models, agents optimize their behavioral policy directly, without ever representing the value of different options. That is, rather than tracking the value of options and using those valuations to compute behavioral policy, these models suggest that agents use the outcomes of different actions to directly update the parameters of the behavioral policy function. This process entails lower computational requirements, but also bears limitations (Mongillo et al. 2014). Because the model directly updates the probability of different options being selected only with new experience, the history of past experiences is not otherwise maintained or referenced by this model. Therefore, the passage of time during a test delay (where animals do not gain new experience) should produce no changes in preference. If the test delay *is* treated as new experience in some way, it is possible that the lack of rewards might drive animals to indifference, but such models cannot account for the dramatic preference reversals observed in previous work on the TWR. Because the experiments of the previous study (Van Allsburg and Shahan 2024) failed to observe similar preference reversals, it is possible that these policy gradient models could provide an effective account of choice over this longer timescale. Their application to the experimental context of the previous study (Van Allsburg and Shahan 2024), however, is conceptually fraught. Such models, like explore/exploit models, are typically applied to discrete-trial settings over shorter timescales (see Bennett et al. 2021), so application to a timescale of days and weeks requires some conceptual leaps in generalization, such as linking parameters to the passage of time in a way not suggested by the studies supporting these models.

Regardless, this study’s unexpected results identify an opportunity. This thorough and well-powered, but inconclusive investigation of dynamic averaging has revealed crucial gaps in our understanding of how animals make decisions over time. Knowledge of these gaps may provide the foundation for developing a theory that can provide a more complete description of choice on this timescale and a better understanding of behavior, generally.

## Data Availability

Data and other study materials are available upon request from the corresponding author.

## Appendix

This appendix provides additional mathematical details of the experimental procedure and analysis, including explanations of the models included in the model fit comparison, with citations for more detailed explanations of the theory underlying each model.

### Generating variable-interval schedules using the Fleshler-Hoffman distribution

The Fleshler-Hoffman (1962) distribution provides a method for generating a set of exponentially distributed intervals for variable interval schedules while avoiding extremely short or long values which could produce irregular behavior. In the current study, ten values were derived from this distribution using a mean of 1 (0.052, 0.163, 0.288, 0.432, 0.599, 0.801, 1.053, 1.393, 1.916, and 3.303). These values were then multiplied by the desired mean length of variable interval schedule (e.g. 10 s for a VI-10 s) to produce a set of exponentially distributed intervals centered on that mean (e.g., producing the following set of intervals for a VI-10 s: 0.52 s, 1.63 s, 2.88 s, 4.32 s, 5.99 s, 8.01 s, 10.53 s, 13.93 s, 19.16 s, and 33.03 s). Each option was then randomly assigned one of these ten intervals each time the VI schedule was activated.

### Models included in fit comparison

#### EWMA (Single-timescale exponentially weighted moving average)

EWMAs are a widely used statistical method of dynamic averaging (full description in Killeen 1984). For an EWMA, value (*V*) for a given option at the current moment (*V*_*n*_) is described by Eq. 4.

4 $$V_{n} = \beta q_{n} + \, \left( {{1 }{-}\beta } \right)V_{{n \, {-} \, 1}}$$

The free parameter* β*, is a learning rate which assigns a proportion of weight to rewards received in the current experience (*q*_*n*_). The remainder of weight (1 – *β*) is assigned to past experience, here represented as *V*_*n-1*_, the valuation from the model’s previous update. Valuation is maintained as a single running average, updated recursively. These valuations can then be inserted into the matching law equation as a decision rule, such that the probability of selecting option *A* (*Pr*_*A*_) among two options, *A* and *B*, is equal to *A*’s relative value: *Pr*_*A*_ = *V*_*A*_ / (*V*_*A*_ + *V*_*B*_).

#### EWMA2 (Two-timescale exponentially weighted moving average)

A different approach to dynamic averaging, assigning weight between multiple EWMA integrators averaging over different timescales (different learning rates) can describe more complex effects on preference like SRC (full description in Iigaya et al. 2019). This specific model contains two EWMA integrators (e.g. integrators *1* and *2*), each calculating value recursively using Eq. 2. To calculate value at a given moment, weight is assigned to the two (e.g. *V*_*n1*_ from integrator* 1* and* V*_*n2*_ from integrator *2*) at that moment by an additional parameter (*w*_*1*_), assigning some weight to integrator 1 and the remainder to integrator 2—as shown in Eq. 5.

5 $$V_{n} = \, w_{1} V_{n1} + \left( {{1 }{-}w_{1} } \right)V_{n2}$$

The learning rates for the two integrators are free parameters, as is the weight parameter. The valuations from Eq. 3 for different options can then be inputted to the matching law equation as a decision rule, as in the single-integrator EWMA model. This model therefore has three parameters, all bounded by 0 and 1.

#### EWMA3 (Three-timescale exponentially weighted moving average)

Similarly to EWMA2, the EWMA3 model uses multiple EWMA integrators to calculate valuation over multiple timescales (full description in Iigaya et al. 2019). EWMA3 contains three EWMA integrators (e.g. integrator *1*, *2* and* 3*), each calculating value recursively using Eq. 2. To calculate value at a given moment, weight is assigned to the three valuations (e.g. *V*_*n1*_ from integrator* 1*, etc.) at that moment by two additional parameters, as shown in Eq. 6. The first weight parameter (*w*_*1*_) determines weight given to the valuation of integrator *1*, while the second weight parameter (*w*_*2*_) determines weight given to the valuation of integrator *2,* with the remainder of the weight (1 – *w*_*1*_ – *w*_*2*_) assigned to the valuation of integrator *3*.

6 $$V_{n} = \, w_{1} V_{n1} + w_{2} V_{n2} + \left( {{1 }{-}w_{1} {-}w_{2} } \right)V_{n3} V_{n} = \, w_{1} V_{n1} + \left( {{1 }{-}w_{1} } \right)V_{n2}$$

The learning rates for the three integrators are free parameters, as are the two weight parameters. The valuations from Eq. 4 for different options can then be inputted to the matching law equation as a decision rule, as in the single-integrator EWMA model. This model therefore has five parameters, all bounded by 0 and 1.

#### TWR (The Temporal Weighting Rule)

The Temporal Weighting Rule uses the relative recency of experience as the basis for its weighting of past reward information (full description in Devenport and Devenport 1994). Equation 7 provides the weight for a given experience (*W*_*x*_), according to the TWR. The numerator calculates the experience’s recency as the reciprocal of *t*_*x*_ (time elapsed since that experience), while the denominator sums the recencies of all *n*_*k*_ experiences with that alternative.

7 $$W_{x} = \frac{{1/t_{x} }}{{\mathop \sum \nolimits_{k = 1}^{n} 1/t_{k} }}$$

Each experience is therefore weighted by its *relative* recency—the ratio of each experience’s specific recency to the summed recencies of all the animal’s experiences with that alternative. These weights then factor into the valuation of an alternative (Eq. 8): value (*V*) equals the sum of rewards during each experience (*q*_*x*_) multiplied by the corresponding weight of that experience (*w*_*x*_).

8 $$V = \mathop \sum \limits_{x} w_{x} q_{x}$$

Like valuations from an EWMA, these values are then used as inputs to the matching law as a decision rule. This approach to discounting past experience means that without new experience, the passage of time should cause the value of each option to regress to its overall average. Since weights are based on recency (1/*t*_*x*_), the passage of time (increasing *t*) causes them to decrease hyperbolically.

#### sTWR-fp (Free-parameter scaled Temporal Weighting Rule)

Since the TWR, in its original formulation (Devenport and Devenport 1994) has no parameters to account for differences in how much relative weight is given to more recent experiences, the TWR has been reformulated as the scaled temporal weighting rule (sTWR; full description in Shahan and Craig 2017) (Eq. 9).

9 $$W_{x} = \frac{{1/t_{x}^{c} }}{{\mathop \sum \nolimits_{k = 1}^{n} 1/t_{k}^{c} }}$$

By raising recencies to the power of *c*, this scalar parameter changes how past experience is integrated to valuations—changing how steeply information decays in weight, which allows the model to be scaled to reflect differences in learning rate or the timescale units on which valuation is calculated. The sTWR-fp treats currency as a free parameter (Devenport et al. 1997).

#### sTWR-rr (Reward-rate scaled Temporal Weighting Rule)

The sTWR-rr is identical to the sTWR-fp model, excepting one specific change—treating the currency parameter as a function of running reward rate for each option (full description in Shahan and Craig 2017). The manner in which *c* is calculated at a given moment is given in Eq. 10, where lambda is a free parameter determining how quickly the currency term increases with running reward rate (*r*, the overall average reward rate in rewards/hr across all the sessions that an option has been available).

10 $$c = \lambda r + 1$$

The calculation of c using this approach is performed separately for each of the options being considered. By linking the overall running average frequency of rewards for an option to the extent to which recent experience is weighed more strongly, options which have produced more rewards will discount past experience more rapidly.

### Reward and time operationalization during delays

For EWMA-based models such as EWMA, EWMA2, and EWMA3, time during test delays (including the test session itself) was operationalized as a period of “zero rewards,” rather than a period of “no new experience.” For an EWMA, this approach to operationalizing time causes absolute valuations to decrease as test delay increases, but does not affect relative valuation between options. For EWMA2 and EWMA3, this approach to operationalization is necessary to produce changes in preference during the test delay—if this time were treated instead as “no new experience,” valuations and relative preference from these models would remain static throughout the delay.

As in previous study of the TWR (e.g. Devenport et al. 1997), time during test delays was operationalized as a period of “no new experience” for the TWR, sTWR-fp, and sTWR-rr. This approach was employed because these TWR-based models are capable of reflecting the passage of time’s effect on valuation through the calculation of weights based on relative recencies of experience, where EWMA-based models require new experience (in this case, experience of “zero rewards” during delay) for valuations or preference to change during the delay. Because the outcome of interest was *initial* preference during the test session (relative responding in the first two minutes of the session), rather than terminal or overall mean preference during the test session, calculation of predicted preference at the occasion of the test did not incorporate the test session experience of “zero rewards” for these models. It should be noted that for the TWR and sTWR-fp, incorporating the test session as an experience of “zero rewards” will result in different absolute valuations of options, but will not produce any change in relative preference between options (the outcome of interest). For the sTWR-rr, treating the test as a session-length experience of zero rewards causes a significant change in both relative and absolute valuations which poorly fit the observed data in all three experiments. To ensure consistency between sTWR-rr and the other two TWR-based models in predicting *initial* preference during test sessions, rewards during the test session were therefore not reflected in valuations or preference from the sTWR-rr.

## Footnotes

1 Other factors, such as the risks of predation or competition, necessarily figure in this decision-making process (Kie 1999; Lima and Dill 1990), but for present purposes, we will focus on how animals make decisions in environments where these risks are not present (or held equal for all options) to determine the specific role of reward variability on decision-making processes.

2 There is stronger empirical support for calculations based on the ratios of valuations (as in the matching law), rather than on differences between valuations (as in explore/exploit models) (Addicott et al. 2017).

3 Because proportional preference data is bounded between 0 and 1, the data were transformed to logits (log odds) for statistical analyses to avoid violating normality assumptions (a review of logits can be found in Cramer 2003). Logits are calculated as the natural log of the ratio of a proportion (*p*) to its complement (1-*p*). Proportions ranging from 0 to 1 range from negative to positive infinity as logits, better approximating a normal distribution of the data.
